# Genome-wide identification of *SrbHLH* transcription factors highlights its potential role in rebaudioside A (RA) biosynthesis in *Stevia rebaudiana*

**DOI:** 10.1186/s12870-023-04353-1

**Published:** 2023-07-06

**Authors:** Yuping Li, Yuan Qiu, Xin Xu, Ming Luo

**Affiliations:** 1 Key Laboratory of South China Agricultural Plant Molecular Analysis and Genetic Improvement & Guangdong Provincial Key Laboratory of Applied Botany, South China Botanical Garden, Chinese Academy of Sciences, Guangzhou, 510650 China; 2 University of Chinese Academy of Sciences, Beijing, 100049 China; 3 College of Life Sciences, Southwest Forestry University, Yunnan, 650224 China

**Keywords:** Terpenoids, bHLH transcription factor, Rebaudioside A, *Stevia rebaudiana*

## Abstract

*Stevia rebaudiana* Bertoni is a valuable medicinal plant and an essential source of natural sweetener, steviol glycosides (SGs), with rebaudioside A (RA) being one of the main components of SGs. *bHLH* transcription factors play a crucial role in plant development and secondary metabolism. In this study, 159 *SrbHLH* genes were identified from the *S. rebaudiana* genome, and each gene was named based on its chromosome location. The SrbHLH proteins were then clustered into 18 subfamilies through phylogenetic analysis. The analysis of conserved motifs and gene structure further supported the classification of the SrbHLH family. Chromosomal location and gene duplication events of *SrbHLH* genes were also studied. Moreover, based on the RNA-Seq data of different tissues of *S. rebaudiana*, 28 *SrbHLHs* were co-expressed with structural genes involved in RA biosynthesis. The expression pattern of candidate *SrbHLH* genes were confirmed by qPCR. Finally, dual luciferase reporter assays (DLAs) and subcellular localization analysis verified SrbHLH22, SrbHLH111, SrbHLH126, SrbHLH142, and SrbHLH152 are critical regulators of RA biosynthesis. This study provides new insights into the function of *SrbHLHs* in regulating SGs biosynthesis and lays the foundation for future applications of *SrbHLH* genes in molecular breeding of *S. rebaudiana*.

**Supplementary Information:**

The online version contains supplementary material available at 10.1186/s12870-023-04353-1.

## Introduction

Transcription factors (TFs) are crucial in regulating various biological processes, including plant development, flowering, metabolism, and abiotic stress responsiveness [1, 2]. Basic helix-loop-helix (bHLH) is one of the most prominent families of transcription factors, and these transcription factors are widely distributed in both plants and animals [3]. The conserved bHLH domain consists of an essential DNA-binding region and two amphipathic *α*-helices separated by a loop of variable length (HLH) [4]. Based on conserved domains and phylogenetic relationships, bHLH can be divided into 15 to 25 subfamilies [5].

In recent years, with the development of whole genome sequencing, numerous *bHLH* gene families have been identified and analyzed in various plant species, such as *Arabidopsis* [6], rice [7], *Erigeron breviscapus* [8], *Aquilaria sinensis* [9], and *Tartary buckwheat* [10]. Many *bHLH* genes have been reported to be involved in regulating terpenoid biosynthesis in plants. For instance, *Catharanthus roseus* CrMYCl or *Artemisia annua* AaMYC2-like proteins regulate the expression of structural genes by binding to the G-box of their promoter regions, thereby affecting the accumulation of indole alkaloids or artemisinins, respectively [11, 12]. Meanwhile, *Medicago truncatula* TSAR1 and TSAR2 regulate the biosynthesis of triterpene saponins by binding to the N-box in the *MtHMGR1* promoter region [13]. Additionally, some key transcription factors can regulate the expression of multiple structural genes in the same metabolic pathway, thereby affecting the accumulation of metabolites. For example, the bHLH transcription factors Bl (Bitter leaf) and Bt (Bitter fruit) in cucumber (*Cucumis sativus*) can bind to the promoters of *oxosqualene cyclase*, *fatty acyltransferase*, and *cytochrome P450* genes for bitter C synthesis simultaneously, regulating the accumulation of bitter taste in cucumber leaves and fruits [14].

*S. rebaudiana* is a medicinal herb with a sweet taste and is an essential source of steviol glycosides (SGs), which are up to three hundred times sweeter than sucrose [15] and have potential applications in the control of diseases such as diabetes, obesity, or hypertension [16]. The biosynthesis of SGs involve two processes of backbone formation and glycosylation [17], with kaurene synthase (*ent*-KS), kaurene oxidase (*ent*-KO), and kaurenoic acid 13-hydroxylase (*ent*-KAH) catalyzing the formation of the backbone, while UDP glycosyltransferases (UGTs) are responsible for glycosylation at the C13-hydroxyl and C19-carboxylic acid positions of the skeleton [17, 18]. Among these SGs, stevioside (ST) and rebaudioside A (RA) are the main components, with RA being more valuable due to its high sweetness and good taste [19]. To date, four *UGT* genes, *UGT74G1*, *UGT85C2*, *UGT91D2*, and *UGT76G1*, involved in RA biosynthesis have been identified [17]. However, the molecular regulatory mechanisms of SG synthesis are poorly understood.

This study aimed to identify and characterize the bHLH gene family in the *S. rebaudiana* genome; the construction of phylogenetic tree was conducted to confirm the relationships between SrbHLH and AtbHLH proteins, the identification of segmentally duplication events were proceeded to explain the expansion of SrbHLH family. We also conducted co-expression analysis between *SrbHLH* genes and structural genes involved in RA synthesis, evaluated the relative expression levels of selected *SrbHLH* genes using qPCR method, and confirmed critical regulators for RA biosynthesis by transient dual luciferase reporter assays (DLA) and subcellular localization analysis. This study provides important clues for future research on the bHLH family in *S. rebaudiana* and sheds light on the molecular regulatory mechanisms underlying SG biosynthesis.

## Methods

### Plant growth and MeJA treatment

*S. rebaudiana* seeds (“Zhongke No. 1” (S1) and “Zhongke No. 4” (S4)) used in this study were identified and planted at South China Botanical Garden, Chinese Academy of Sciences, Guangzhou City, Guangdong Province. All study protocols for this species comply with relevant institutional, national, and international guidelines and legislations. Both S1 and S4 varieties were used for tissue expression analysis, and S4 was used for MeJA treatment experiments.

After removing the villi from the seed surface, the seeds were soaked in ddH_2_O for 2 h; Then, floating seeds were removed, and the remaining plump seeds were sterilized using 1% NaClO solution for 10 min; Furthermore, the sterilized seeds were washed 5 times with sterilized ddH_2_O, planted on MS plates, and moved to a culture room with a temperature of 25 ℃ and a photoperiod cycle of 16 h of light and 8 h of dark.

For the MeJA treatment experiment, 1-week-old seedlings were transferred to MS plates with 100 μM MeJA. After 6 h, 12 h, and 24 h, leaves from 30 seedlings were harvested and pooled as one sample, then immediately frozen with liquid nitrogen and stored at -80˚C. All experiments were performed with three biological replicates.

### Identification of *bHLH* genes in the *S. rebaudiana* genome

The *S. rebaudiana* genome was sequenced and assembled in our laboratory. The Hidden Markov model (HMM) file of the bHLH domain (PF00010) was downloaded from the Pfam database (http://pfam.xfam.org/). Using PF00010 as bait, the *SrbHLH* gene family members were identified by searching the *S. rebaudiana* genome with the hmmsearch program (HMMER 3.1b1). To further verify the existence of the bHLH conserved domain, the PFAM and SMART (http://smart.emblheidelberg.de/) programs were used. Finally, the amino acid lengths, molecular weights, and isoelectric points of candidate SrbHLHs were analyzed via the tools on the ExPASy website (https://www.expasy.org/), and subcellular localizations of SrbHLH proteins were predicted using the WoLF PSORT website (https://wolfpsort.hgc.jp/).

### Gene structure, genome distribution, and phylogenetic analysis

The generic feature format (GFF) file of the *S. rebaudiana* genome was used to obtain the gene structure and chromosomal location information of each SrbHLH. The conserved motif of each SrbHLH was predicted using the MEME web tool (https://meme-suite.org/meme/), and the gene structures were displayed using TBtools [20].

To construct the phylogenetic tree, protein sequences of *Arabidopsis* were downloaded from the Phytozome database (https://phytozome-next.jgi.doe.gov/). The bHLH proteins from *Arabidopsis* and *S. rebaudiana* were aligned using the ClustalW program, the Neighbor-Joining (NJ) method was used in MEGA 7.0, and 1000 bootstrap replicates were performed using the SrbHLH and AtbHLH proteins.

### RNA-Seq co-expression analysis and candidate selection

Using RNA-Seq data from different tissues of two *S. rebaudiana* varieties, we identified enzyme-coding structural genes involved in RA biosynthesis, including *ent-KS*, *ent-KO*, *ent-KAH*, *UGT74G1*, *UGT85C2*, *UGT91D2*, and *UGT76G1*. These structural genes were used as bait to screen bHLH transcription factors that potentially regulate RA biosynthesis. Co-expression relationships between structural genes and *SrbHLHs* were estimated using Pearson correlation analysis. We filtered a set of co-expressed *SrbHLHs* genes using a Pearson coefficient threshold of 0.95 and *p* < 0.05. The OmicShare tools, a free online platform for data analysis (https://www.omicshare.com/tools), were applied to construct the *structural-SrbHLH* gene network.

### RNA extraction and qPCR analysis

Total RNA extraction was conducted using a HiPure Total RNA Mini Kit (Code No. R4151-03). Vazyme HiScript Reverse Transcriptase (Cat No. R101-01) was used for cDNA synthesis using the manufacturer’s instructions. qPCR was performed in 384-well plates using Vazyme HiScript Q RT SuperMix (Code No. R122-01) with a LightCycler 480 (Roche, Switzerland). The expression levels of each tested gene relative to the internal reference gene (Sractin) were determined using the 2^−ΔΔCT^ method [21]. All primers used in this study are listed in Table S1. Three biological replicates and three technical replicates were conducted.

### Transient dual luciferase reporter assays

The promoter sequence (1500 bp of upstream of the transcription start site) of *UGT76G1* was cloned into the pGreen II 0800-LUC reporter vector, and the full length coding sequence of *SrbHLHs* was cloned into the pCambia1300-UBQ-GFP effector vector. The constructed effector and reporter plasmids were transformed into *Agrobacterium* GV3101 and injected into tobacco leaves (*Nicotiana benthamiana* Domin) alongside an agrobacterium-mediated transient expression system [22]. An empty pCambia1300-UBQ-GFP vector was used as a negative control. Firefly and *Renilla* luciferase activity was measured using a dual luciferase reporter assay system (Promega, Madison, WI, USA). Results were evaluated as the ratio of firefly luciferase (LUC) to *Renilla* luciferase (REN) activity across three independent biological replicates. Primers for vector construction are listed in Supplemental Table S1.

### Subcellular localization assays in tobacco leaves

The subcellular localizations of SrbHLHs were verified through transient expression assays in *N. benthamiana* leaves. The full-length CDSs of candidate SrbHLHs were cloned into pCambia1300-UBQ-GFP in-frame with a GFP reporter. The primers are listed in Table S1. The fusion constructs were co-transfected into *N. benthamiana* leaves alongside a nuclear localization marker (Histone3-mCherry). After incubation for 3 days, the localization of the target proteins was observed using a confocal fluorescence microscope (Leica, SP8).

## Results

### Identification of the *SrbHLH* gene family in *S. rebaudiana*

After using NCBI Batch CD-Search tools to eliminate redundant proteins, we obtained 159 bHLHs from the *S. rebaudiana* genome. To distinguish these genes, we named them SrbHLH1 to SrbHLH159 based on their location on the chromosomes of *S. rebaudiana* (Table 1). Next, we examined various physicochemical properties of the SrbHLHs, including amino acid lengths, isoelectric point (PI), protein molecular weight (MW), and subcellular localization. Among the 159 SrbHLH proteins, their amino acid sequences varied from 93 aa (SrbHLH44) to 661 aa (SrbHLH35). The MWs of SrbHLH proteins ranged from 10.33 kDa to 70.78 kDa, while the PIs of the SrbHLH proteins ranged from 4.38 (SrbHLH128) to 10.15 (SrbHLH159). Additionally, based on subcellular localization predictions, most of the SrbHLH proteins were expected to be located in the nucleus, cytoplasm, or chloroplast, with only SrbHLH34 likely found in the cytoskeleton (Table 1).

**Table 1:** Detailed information of bHLH members in *S. rebaudiana*

**Gene id**	**Name**	**Location**	**Amino acid lengths**	**MW (KDa)**	**pI**	**Subcellular localization**
Str01G020234.g	SrbHLH1	Chr01:10300857-10302344	258	29.05	8.64	nucl,chlo,cyto,extr
Str01G021361.g	SrbHLH2	Chr01:36803643-36806436	292	31.77	5.15	nucl,plas,cysk
Str01G022283.g	SrbHLH3	Chr01:50967180-50970396	425	47.15	6.80	nucl
Str01G022296.g	SrbHLH4	Chr01:53186930-53190132	468	51.88	6.59	nucl,chlo
Str01G022719.g	SrbHLH5	Chr01:61053132-61056914	336	37.18	4.74	cyto,golg,plas,ER_plas
Str01G022740.g	SrbHLH6	Chr01:62646457-62647737	240	26.90	8.16	chlo,mito,cyto,plas
Str01G022825.g	SrbHLH7	Chr01:62286722-62288492	264	30.13	6.63	nucl,cyto_nucl,cysk,cyto
Str01G022826.g	SrbHLH8	Chr01:62293918-62294801	211	24.30	8.10	nucl,cyto,chlo,mito
Str01G023178.g	SrbHLH9	Chr01:68563478-68565564	276	32.21	8.37	nucl,extr
Str01G023257.g	SrbHLH10	Chr01:69897012-69900845	232	26.55	5.24	nucl
Str01G023566.g	SrbHLH11	Chr01:77437443-77439928	164	18.58	8.51	nucl,cyto
Str01G023583.g	SrbHLH12	Chr01:76493689-76496169	258	28.93	6.80	nucl,cyto_nucl,chlo,mito
Str01G023779.g	SrbHLH13	Chr01:78455977-78458858	467	52.02	6.15	nucl
Str01G024023.g	SrbHLH14	Chr01:83718205-83721178	243	26.57	6.79	nucl
Str01G024065.g	SrbHLH15	Chr01:87374661-87377167	198	21.38	9.25	nucl
Str01G024104.g	SrbHLH16	Chr01:87871606-87872583	215	23.92	9.74	nucl
Str01G024108.g	SrbHLH17	Chr01:88676115-88680015	329	37.48	5.51	nucl,cyto,extr,cysk
Str01G024191.g	SrbHLH18	Chr01:87268019-87273710	291	32.58	7.47	nucl,cyto,cysk,plas
Str01G024207.g	SrbHLH19	Chr01:87762590-87766808	322	35.75	7.98	nucl,cyto,cysk
Str01G024258.g	SrbHLH20	Chr01:88292483-88295991	381	39.49	5.81	nucl,plas,cysk
Str01G024316.g	SrbHLH21	Chr01:90332046-90335507	509	55.51	7.35	nucl,pero
Str01G024342.g	SrbHLH22	Chr01:91373043-91377473	489	53.49	7.05	nucl
Str01G024409.g	SrbHLH23	Chr01:90588331-90595596	484	55.34	4.72	nucl,vacu
Str01G024555.g	SrbHLH24	Chr01:95152705-95155568	322	36.29	5.42	nucl,cyto,extr
Str01G024823.g	SrbHLH25	Chr01:101488556-101493787	313	34.12	4.90	nucl,cyto_nucl,extr
Str01G025225.g	SrbHLH26	Chr01:108102493-108105958	459	51.11	7.46	nucl
Str01G026388.g	SrbHLH27	Chr01:132201062-132204047	212	23.90	5.85	nucl,chlo,cysk
Str01G026511.g	SrbHLH28	Chr01:128803794-128805235	160	18.02	8.68	nucl,cyto,cysk
Str01G026534.g	SrbHLH29	Chr01:134641111-134646093	373	42.34	6.44	nucl
Str01G026555.g	SrbHLH30	Chr01:134695706-134702169	333	37.67	7.03	nucl,extr
Str01G026605.g	SrbHLH31	Chr01:134483978-134486955	314	35.57	5.11	nucl
Str01G027358.g	SrbHLH32	Chr01:172487533-172489201	263	29.83	8.24	chlo,vacu,nucl,cyto
Str01G027359.g	SrbHLH33	Chr01:172445716-172447234	192	22.21	9.95	cyto,cyto_nucl,nucl,plas
Str01G027471.g	SrbHLH34	Chr01:174245780-174249887	435	48.41	6.98	nucl,golg,cyto,vacu
Str01G027585.g	SrbHLH35	Chr01:179512403-179521226	661	70.78	8.07	nucl,extr
Str01G027603.g	SrbHLH36	Chr01:178859836-178862817	379	42.10	6.09	nucl,cyto_nucl,cyto,chlo
Str01G027617.g	SrbHLH37	Chr01:180585446-180589879	282	32.35	5.03	cyto,nucl,plas
Str01G027799.g	SrbHLH38	Chr01:180864922-180869069	307	32.31	6.10	nucl,cyto,plas
Str01G027939.g	SrbHLH39	Chr01:186902476-186904880	244	27.23	6.05	nucl,cyto,cysk
Str01G028237.g	SrbHLH40	Chr01:186551294-186553567	318	31.26	5.33	nucl,chlo
Str02G028524.g	SrbHLH41	Chr02:4678281-4679696	267	28.70	6.53	nucl,cyto_nucl
Str02G028525.g	SrbHLH42	Chr02:4636467-4637827	248	26.60	5.43	nucl
Str02G028707.g	SrbHLH43	Chr02:5205852-5207554	289	32.47	6.95	cysk
Str02G028858.g	SrbHLH44	Chr02:8084206-8085614	93	10.33	9.31	nucl,mito,cyto_nucl,chlo
Str02G028945.g	SrbHLH45	Chr02:8879524-8881211	273	30.04	6.53	nucl
Str02G029578.g	SrbHLH46	Chr02:21123197-21125604	410	46.66	8.06	nucl,cyto,chlo,plas
Str02G030121.g	SrbHLH47	Chr02:36769105-36771796	347	37.09	6.60	nucl
Str02G030266.g	SrbHLH48	Chr02:39827283-39829976	347	37.09	6.60	nucl
Str02G030268.g	SrbHLH49	Chr02:39798845-39801534	246	26.66	5.38	nucl
Str02G031191.g	SrbHLH50	Chr02:93307670-93312225	365	40.44	7.46	chlo,nucl
Str02G031980.g	SrbHLH51	Chr02:124111427-124119614	523	58.50	8.21	nucl,cyto,mito,pero
Str02G032795.g	SrbHLH52	Chr02:150352939-150355935	332	37.06	7.86	nucl,pero
Str02G032798.g	SrbHLH53	Chr02:150359809-150362202	274	30.70	9.08	nucl,extr
Str02G032881.g	SrbHLH54	Chr02:152948154-152950336	326	36.61	8.04	nucl,cyto_nucl,cyto,chlo
Str02G033790.g	SrbHLH55	Chr02:170351359-170352817	281	31.33	9.50	nucl,cyto_nucl,cyto,chlo
Str02G033903.g	SrbHLH56	Chr02:171052414-171053873	246	27.47	7.91	nucl,cyto_nucl,cyto,extr
Str02G034059.g	SrbHLH57	Chr02:173377309-173387571	294	33.51	6.59	nucl,cyto,extr,cysk
Str02G034426.g	SrbHLH58	Chr02:180720924-180722990	314	36.31	4.63	nucl,cyto_nucl,extr
Str02G034437.g	SrbHLH59	Chr02:180654020-180655150	287	33.12	5.07	nucl,extr,cysk
Str02G034476.g	SrbHLH60	Chr02:180429731-180431301	254	29.32	4.80	nucl,cyto_nucl,chlo,golg
Str02G034549.g	SrbHLH61	Chr02:180914176-180915672	329	35.94	5.98	nucl,extr,cyto
Str02G034616.g	SrbHLH62	Chr02:180799840-180801962	299	33.35	6.24	nucl
Str02G034617.g	SrbHLH63	Chr02:180813721-180815862	299	33.35	6.24	nucl
Str02G034970.g	SrbHLH64	Chr02:187340812-187342830	339	37.45	7.79	nucl,cyto,plas
Str02G035141.g	SrbHLH65	Chr02:193438112-193440035	254	28.95	6.36	nucl,cyto_nucl,cyto,cysk
Str02G035179.g	SrbHLH66	Chr02:189956737-189958569	311	34.10	7.42	nucl,plas
Str02G035206.g	SrbHLH67	Chr02:193633004-193635119	300	31.62	6.16	nucl
Str02G035597.g	SrbHLH68	Chr02:197632334-197633754	358	40.60	4.88	nucl,vacu
Str02G035750.g	SrbHLH69	Chr02:199435897-199445235	499	55.30	8.93	nucl,cyto_nucl,cyto,pero
Str02G035799.g	SrbHLH70	Chr02:201562436-201572045	477	52.78	8.70	nucl,cyto_nucl,cyto,pero
Str02G035908.g	SrbHLH71	Chr02:202931924-202933229	225	24.55	8.96	nucl,cyto,chlo
Str02G036012.g	SrbHLH72	Chr02:204287596-204291812	304	34.35	7.13	nucl,cyto,cysk,plas
Str02G036425.g	SrbHLH73	Chr02:211747255-211749798	399	43.75	6.72	nucl,cysk
Str03G039466.g	SrbHLH74	Chr03:17258275-17260947	373	39.81	6.87	nucl
Str03G039581.g	SrbHLH75	Chr03:20098268-20100950	375	40.10	6.76	nucl
Str03G040173.g	SrbHLH76	Chr03:33836720-33846841	268	30.24	8.46	nucl,cyto_nucl,cyto,cysk
Str03G040185.g	SrbHLH77	Chr03:34470952-34480046	320	36.36	7.64	nucl,cyto,chlo,cysk
Str03G041517.g	SrbHLH78	Chr03:126340076-126341460	236	26.72	8.12	cyto,cysk,nucl,ER_vacu
Str03G041801.g	SrbHLH79	Chr03:131382434-131388132	332	37.74	7.84	nucl
Str03G041802.g	SrbHLH80	Chr03:131372062-131376495	333	37.75	7.63	nucl
Str03G041818.g	SrbHLH81	Chr03:132741636-132743642	287	32.07	6.52	nucl,cyto_nucl,chlo,cyto
Str03G041969.g	SrbHLH82	Chr03:135900272-135902828	355	39.20	6.53	nucl,cyto_nucl,cysk,chlo
Str03G042161.g	SrbHLH83	Chr03:137902352-137905917	256	28.99	9.66	pero,cyto,nucl
Str03G042635.g	SrbHLH84	Chr03:144439543-144441256	314	34.79	7.21	nucl,chlo
Str04G047125.g	SrbHLH85	Chr04:21690632-21693905	273	30.60	7.15	nucl
Str04G047272.g	SrbHLH86	Chr04:33710004-33713030	402	45.12	8.25	pero,nucl,cyto
Str04G047412.g	SrbHLH87	Chr04:36980619-36986893	340	38.75	6.65	nucl,cyto,extr
Str04G047494.g	SrbHLH88	Chr04:38454689-38460919	368	41.55	6.16	nucl,cyto,plas,extr
Str04G047650.g	SrbHLH89	Chr04:41729293-41732320	402	45.10	8.25	pero,nucl,cyto
Str04G047731.g	SrbHLH90	Chr04:45420512-45423500	216	23.96	4.86	nucl,cysk,chlo,cyto
Str04G048063.g	SrbHLH91	Chr04:54530219-54532230	490	53.85	5.67	nucl,pero
Str04G048663.g	SrbHLH92	Chr04:64996617-65001790	164	18.92	5.77	nucl,pero
Str04G049508.g	SrbHLH93	Chr04:80231060-80231737	203	22.79	8.30	nucl
Str04G049533.g	SrbHLH94	Chr04:80663210-80663735	156	17.30	9.38	cyto,chlo,nucl
Str04G049551.g	SrbHLH95	Chr04:80837853-80841290	210	22.96	7.32	nucl
Str04G049751.g	SrbHLH96	Chr04:84274149-84275503	321	36.49	4.50	nucl,extr
Str04G049975.g	SrbHLH97	Chr04:87635896-87638727	232	25.80	5.68	nucl,chlo,cyto
Str04G050069.g	SrbHLH98	Chr04:89432360-89433569	208	24.02	9.91	nucl,cyto_nucl,chlo,cysk
Str04G050148.g	SrbHLH99	Chr04:90583436-90584164	215	24.15	6.53	nucl
Str04G050164.g	SrbHLH100	Chr04:90391438-90393343	274	30.29	7.65	nucl,cyto_nucl
Str04G050460.g	SrbHLH101	Chr04:95954004-95956313	407	44.18	8.47	vacu,extr,chlo,nucl
Str04G050582.g	SrbHLH102	Chr04:97404212-97406498	333	37.74	9.10	nucl,vacu,mito_plas
Str04G050623.g	SrbHLH103	Chr04:98704668-98707943	244	26.81	5.41	nucl,cyto_nucl
Str04G050856.g	SrbHLH104	Chr04:101394243-101395939	250	28.40	9.58	nucl,cyto_nucl,chlo,extr
Str04G051075.g	SrbHLH105	Chr04:104184552-104185926	219	25.08	8.33	cyto,nucl,extr
Str05G006744.g	SrbHLH106	Chr05:1847033-1850190	499	54.61	6.26	nucl
Str05G006893.g	SrbHLH107	Chr05:4477024-4478989	269	30.63	5.21	nucl,vacu
Str05G006930.g	SrbHLH108	Chr05:5150084-5152270	247	27.75	7.06	nucl,chlo
Str05G008689.g	SrbHLH109	Chr05:29825921-29828290	199	22.45	4.88	nucl,pero
Str05G008691.g	SrbHLH110	Chr05:29814601-29816552	166	19.15	5.01	nucl,pero
Str05G008814.g	SrbHLH111	Chr05:31320740-31326657	444	47.43	8.09	nucl,cyto
Str05G010651.g	SrbHLH112	Chr05:54162295-54164765	503	52.75	6.84	nucl,cyto
Str05G010698.g	SrbHLH113	Chr05:55234053-55236371	452	48.65	6.96	nucl
Str05G010862.g	SrbHLH114	Chr05:57861289-57863087	333	36.75	5.23	nucl
Str05G011943.g	SrbHLH115	Chr05:79765690-79769104	355	40.25	9.81	nucl,chlo,,
Str05G012785.g	SrbHLH116	Chr05:120636445-120638313	308	34.87	8.10	nucl,cyto_nucl,cyto,chlo
Str06G015950.g	SrbHLH117	Chr06:10396197-10398293	326	31.27	7.94	nucl
Str06G016582.g	SrbHLH118	Chr06:26794592-26798306	340	38.82	5.65	nucl,cyto,extr
Str06G017601.g	SrbHLH119	Chr06:56604048-56610725	285	31.24	8.88	nucl,chlo,cyto,cysk
Str06G017602.g	SrbHLH120	Chr06:56592075-56598813	337	36.75	7.00	nucl,cyto,cysk,chlo
Str06G017952.g	SrbHLH121	Chr06:82957026-82959161	348	36.91	6.80	nucl,chlo,,
Str06G018118.g	SrbHLH122	Chr06:89503376-89507686	350	39.15	8.33	nucl,chlo,mito,plas
Str07G002461.g	SrbHLH123	Chr07:663698-666164	516	55.44	5.16	nucl,pero
Str07G002651.g	SrbHLH124	Chr07:3280217-3282025	602	65.10	5.82	nucl
Str07G002657.g	SrbHLH125	Chr07:3379352-3382091	341	37.09	5.61	nucl,chlo,cyto,extr
Str07G002920.g	SrbHLH126	Chr07:6402147-6404480	462	51.16	7.03	nucl
Str07G002921.g	SrbHLH127	Chr07:6406123-6407400	223	25.11	9.10	nucl,cyto,pero
Str07G003905.g	SrbHLH128	Chr07:20799314-20802263	174	19.96	4.38	nucl
Str07G004042.g	SrbHLH129	Chr07:23401220-23405016	388	40.65	5.91	golg,vacu,nucl,plas
Str07G004204.g	SrbHLH130	Chr07:27077869-27079730	323	36.54	6.87	nucl
Str07G004388.g	SrbHLH131	Chr07:31352730-31355458	238	26.62	6.30	nucl,pero
Str07G004524.g	SrbHLH132	Chr07:33124243-33126796	238	26.62	6.30	nucl,pero
Str07G004769.g	SrbHLH133	Chr07:38214615-38215867	251	27.75	6.14	nucl,cysk
Str07G004805.g	SrbHLH134	Chr07:38539725-38542859	306	34.76	8.47	nucl
Str07G005716.g	SrbHLH135	Chr07:97560643-97564632	532	56.60	6.97	nucl
Str07G005970.g	SrbHLH136	Chr07:106603679-106606760	312	34.99	5.43	nucl,cyto_nucl
Str07G005983.g	SrbHLH137	Chr07:107472505-107475779	320	35.96	5.09	nucl,cyto_nucl
Str07G006434.g	SrbHLH138	Chr07:120405602-120408868	144	16.33	9.45	nucl,cyto_nucl,mito,golg_plas
Str08G012969.g	SrbHLH139	Chr08:13247797-13250106	316		8.03	nucl
Str08G013104.g	SrbHLH140	Chr08:20482583-20489244	228	25.44	9.61	nucl
Str08G013693.g	SrbHLH141	Chr08:49549839-49552461	496	55.71	7.91	nucl,cyto
Str08G013709.g	SrbHLH142	Chr08:50732610-50735231	496	55.69	7.87	nucl,cyto,vacu,cysk
Str08G014470.g	SrbHLH143	Chr08:92815872-92819705	185	20.92	9.04	nucl,cyto,pero,chlo
Str08G014518.g	SrbHLH144	Chr08:95094647-95097345	391	41.69	8.42	nucl
Str09G037585.g	SrbHLH145	Chr09:23178108-23181614	238	26.33	6.29	nucl,pero
Str09G038394.g	SrbHLH146	Chr09:77769981-77773292	373	39.51	5.86	nucl
Str10G043997.g	SrbHLH147	Chr10:17270343-17272849	369	36.60	6.42	nucl
Str10G044315.g	SrbHLH148	Chr10:24249440-24252417	249	27.92	7.76	nucl,chlo,extr,cysk
Str10G044805.g	SrbHLH149	Chr10:36291843-36293075	160	17.81	9.02	nucl,chlo,cyto,mito
Str10G045552.g	SrbHLH150	Chr10:78940059-78941461	254	27.26	6.89	nucl,pero
Str10G045587.g	SrbHLH151	Chr10:80941007-80942402	213	24.26	7.75	nucl,pero
Str10G046000.g	SrbHLH152	Chr10:93207551-93209448	426	45.93	5.90	nucl
Str10G046733.g	SrbHLH153	Chr10:108224452-108226955	220	24.86	5.05	nucl,pero
Str10G046798.g	SrbHLH154	Chr10:113370534-113373852	456	45.35	7.92	nucl
Str10G046808.g	SrbHLH155	Chr10:110977219-110980492	446	44.16	8.70	nucl
Str11G000165.g	SrbHLH156	Chr11:3600292-3602621	150	16.98	8.65	chlo,mito,cyto_nucl,nucl
Str11G000571.g	SrbHLH157	Chr11:14444180-14448295	451	47.73	5.08	nucl
Str11G001423.g	SrbHLH158	Chr11:53610340-53612965	380	42.43	5.61	nucl,cyto,extr,cysk
Str11G001429.g	SrbHLH159	Chr11:53818316-53822900	202	22.77	10.15	nucl,nucl_plas,chlo,cyto

### Phylogenetic analysis and classification of *SrbHLH* genes

**Fig.1:**
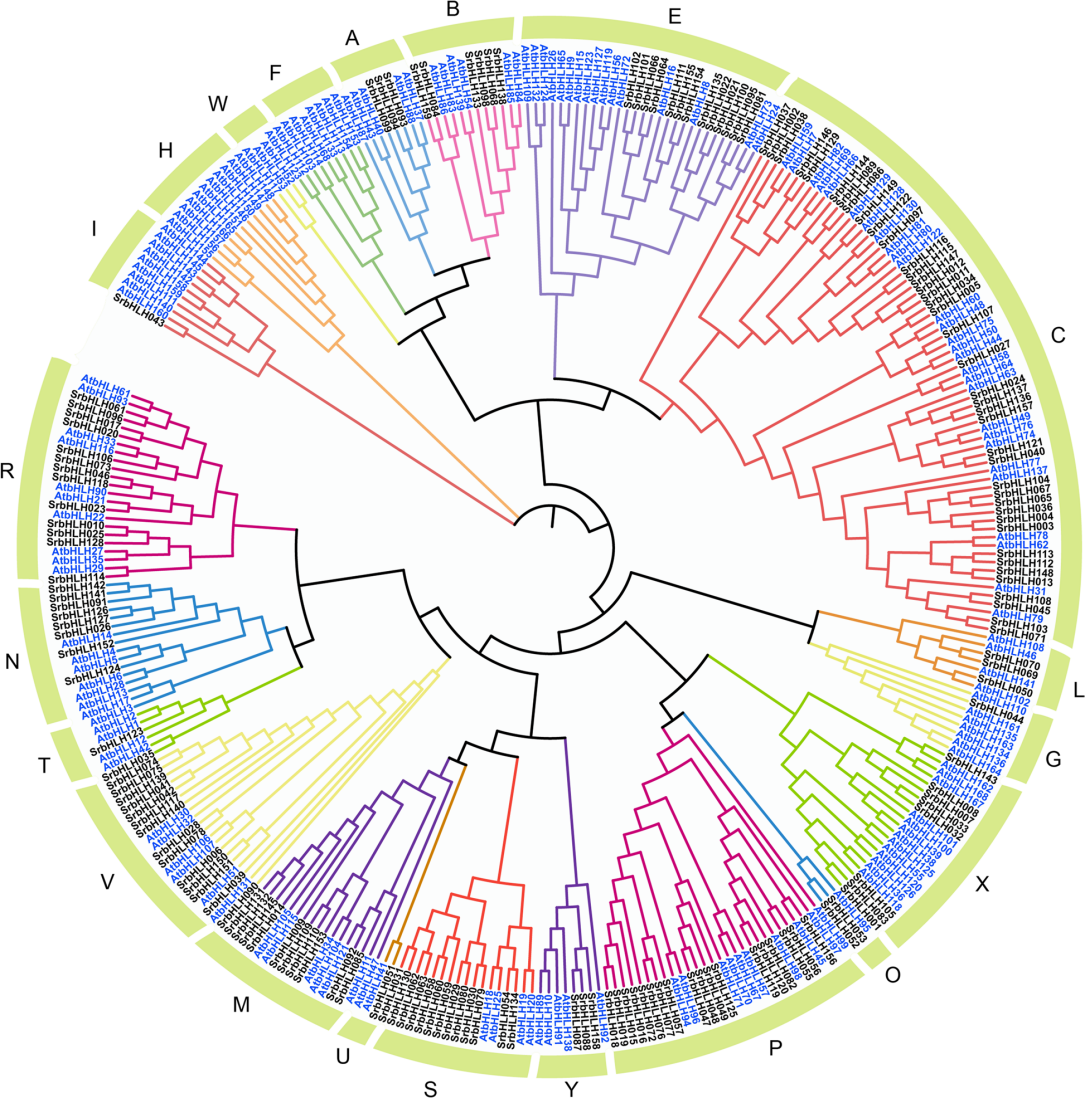
Phylogenetic tree of bHLH proteins in *S. rebaudiana* and *Arabidopsis thaliana*. The phylogenetic tree was obtained using the NJ method in MEGA7. The tree shows 21 phylogenetic subfamilies, each subfamily represented by different colors. bHLH proteins from *Arabidopsis* are labeled in blue

To evaluate the classification and evolutionary relationships among SrbHLHs in plants, we utilized bHLH amino acid sequences from *S. rebaudiana* and *Arabidopsis* to construct a Neighbor-Joining (NJ) tree using MEGA 7.0 software. The phylogenetic analysis results revealed that 159 SrbHLHs were categorized into 18 subfamilies (Fig. 1), including subfamilies A, B, C, E, G, I, L, M, N, O, P, R, S, T, U, V, X, and Y, consistent with previous research [7, 8]. Subfamily C contained 40 SrbHLHs, while subfamily P and subfamily V contained 18 and 14 SrbHLHs, respectively. Furthermore, subfamily E, S, and R included 13 SrbHLHs, whereas subfamily I, subfamily G, subfamily U, and subfamily T had only one assigned SrbHLH. Notably, subfamilies H, W, and F had no SrbHLHs assigned to them.

### Gene structure and motif analysis of *SrbHLH* genes in *S. rebaudiana*

**Fig. 2:**
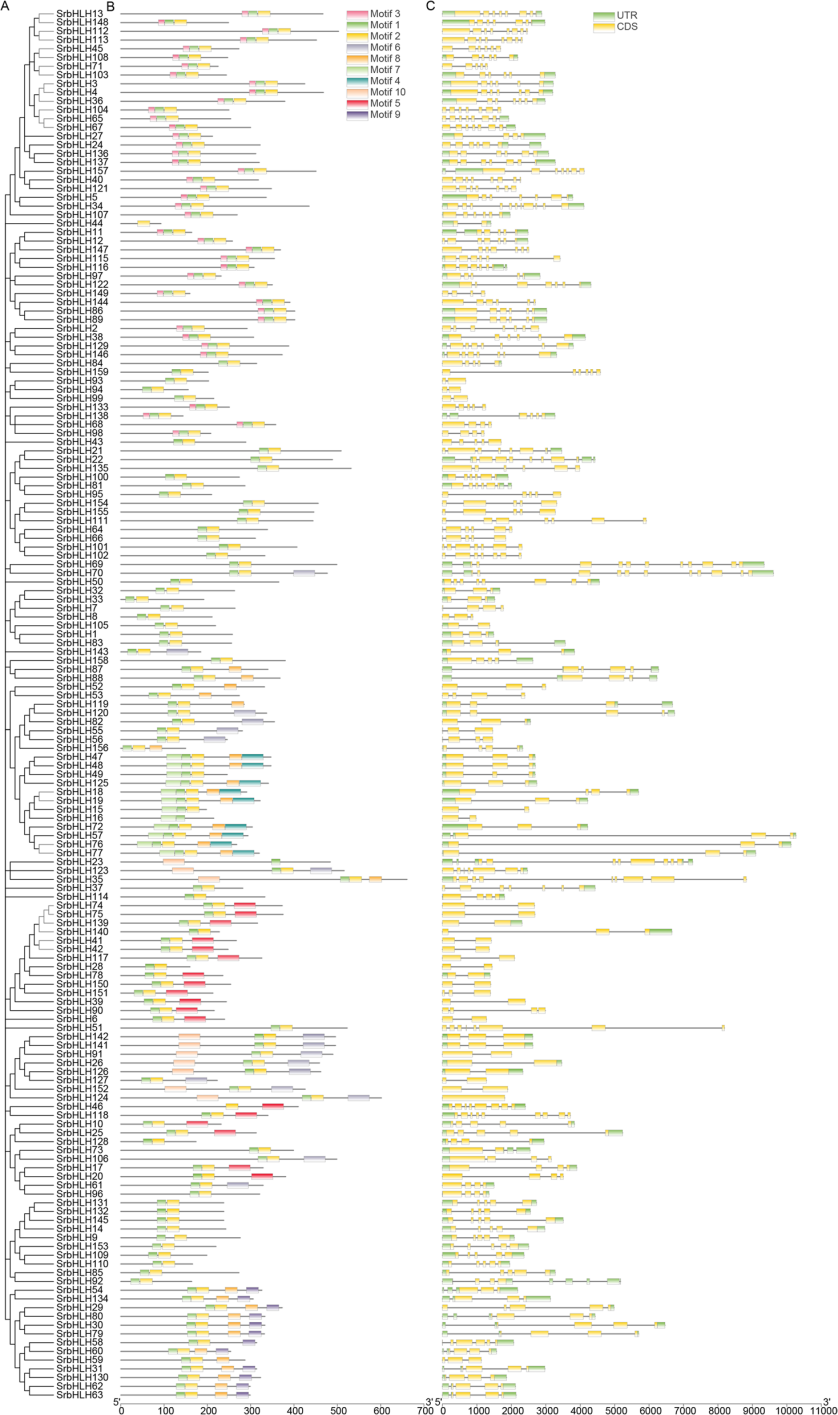
The gene structure and motif distribution analysis of the SrbHLH proteins in *S. rebaudiana*. **A** Phylogenetic trees of SrbHLH proteins constructed by the NJ method; **B** Ten conserved motifs in the SrbHLH protein; **C** Exon and intron distribution of *SrbHLH* genes, green rectangles, and gray lines show the exons and introns, respectively

To investigate the characteristic regions of SrbHLH proteins, we utilized MEME to identify conserved motifs among the 159 SrbHLH proteins (Fig. 2). Ten distinct motifs were identified and designated as motif 1 through 10. Figure 2A illustrates that SrbHLH proteins contain different numbers of conserved motifs ranging from 1 to 5. Almost all SrbHLH family members contain two highly conserved motifs (motif 1 and 2). Additionally, we observed that SrbHLH proteins belonging to the same subfamily typically share similar composition and relative position. For instance, SrbHLH proteins within subfamily P contain motif 1, motif 2, motif 4, motif 7, and motif 8, whereas the members of subfamily V share common motifs 1, 2, and 5 (Fig. 2A). The fact that members of the same subfamily have similar gene structure and motif composition and tend to gather together in the phylogenetic trees supports the accuracy and authenticity of the subfamily classification.

To explore their structural composition, we further analyzed the UTR, exons, and introns structure of identified SrbHLH genes (Fig. 2B). By comparing the number and position of exons and introns, we observed that the 159 *SrbHLH* genes possess varying numbers, ranging from 1 to 11, with only one *SrbHLH* gene being intronless. Some subfamilies contained genes with a specific number of exons, such as subfamily A, where 3 *SrbHLH* genes in this clade contained 2 exons. Generally, genes clustered into the same group in the phylogenetic tree have a similar number of exons, such as *SrbHLH47*, *SrbHLH48*, *SrbHLH49*, and *SrbHLH125*, which each contain three exons, with similar relative positions to the UTR.

### Chromosome distribution, gene duplication, and synteny analysis of *SrbHLH* genes in the *S. rebaudiana* genome

**Fig. 3:**
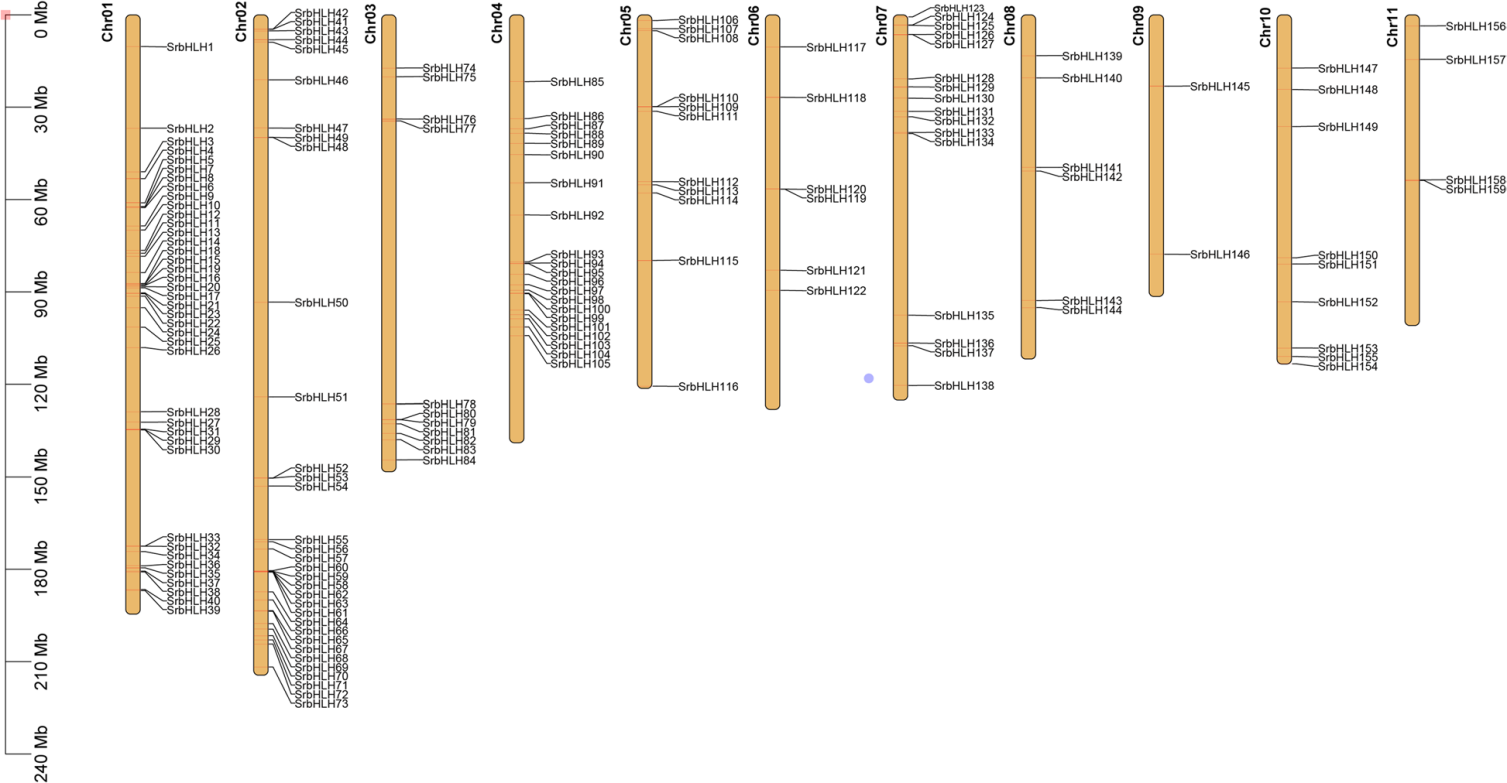
Chromosome distribution of *SrbHLH* genes in *S. rebaudiana* genome. The chromosome numbers are shown on the top of each chromosome

TBtools was utilized to investigate the regions of interest for *SrbHLH* genes on the chromosome. The analysis involved anchoring 159 *SrbHLH* genes to their corresponding chromosomes. The results demonstrated that the distribution of *SrbHLH* genes on the *S. rebaudiana* genome was irregular, and the genes were randomly located on all 11 chromosomes (Fig. 3). Chromosome 1 had the most *SrbHLHs*, with 40 genes, followed by chromosome 2, which had 33 genes. Conversely, chromosome 9 had the least *SrbHLHs*, with only 2 genes.

**Fig. 4:**
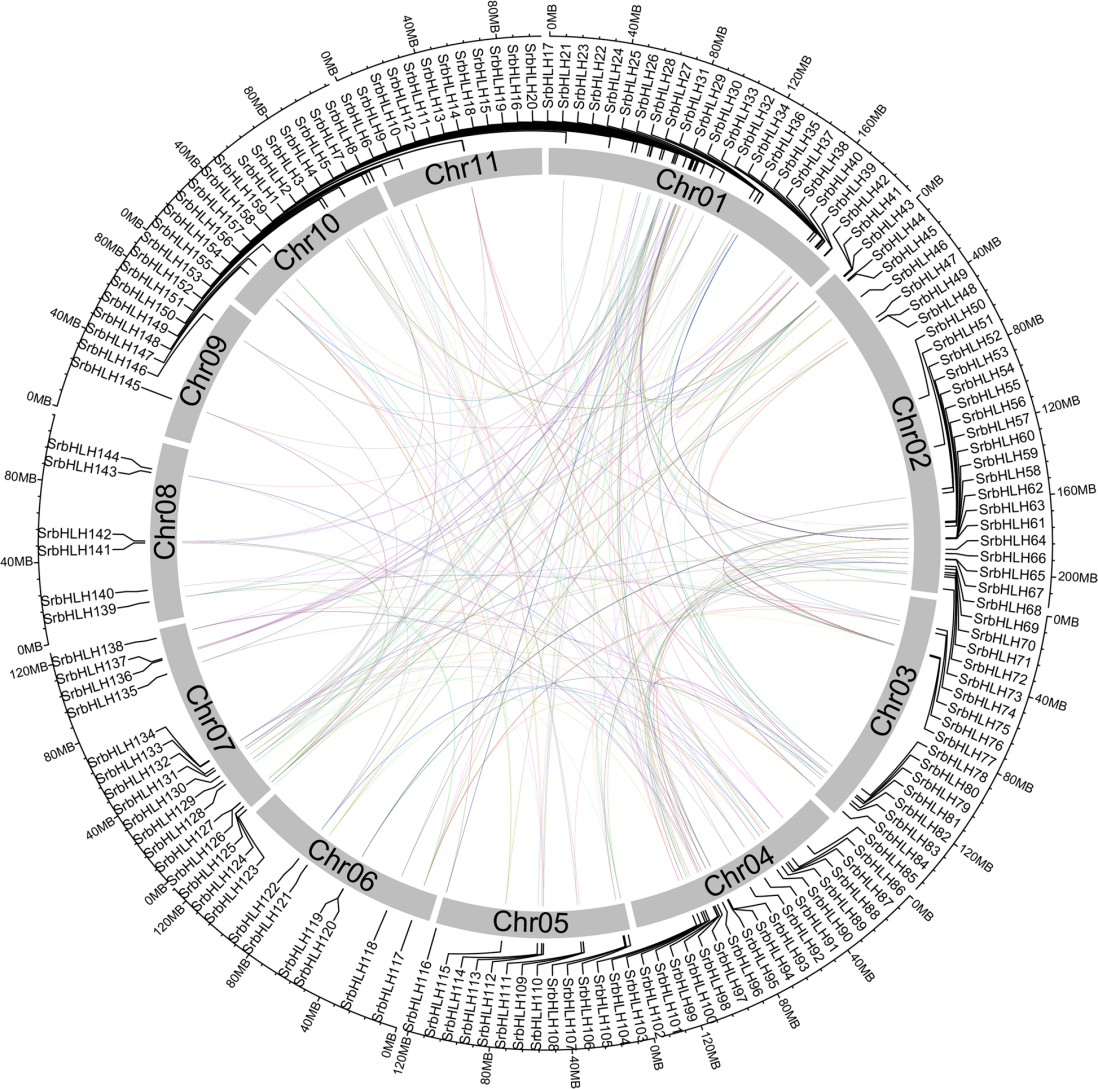
Collinearity analysis of the *SrbHLHs* in the *S. rebaudiana* genome. The duplicated *SrbHLHs* were mapped to different chromosomes using shinyCircos. Colorful lines link the collinear relationships among SrbHLHs, and grey boxes present the chromosome

Genome duplication events are considered primary drivers of genome evolution and gene family expansion, with tandem and segmental duplication being the primary patterns. In this study, we observed no tandem repeat events in the SrbHLH family. Still, we did identify 165 pairs of segmental duplicates that were unevenly distributed on different chromosomes in the *S. rebaudiana* genome (Fig. 4). Among these pairs of collinear relationships, some *SrbHLHs* were paired with more than one gene. For instance, *SrbHLH149* had a collinear relationship with *SrbHLH11*, *SrbHLH97*, *SrbHLH115* and *SrbHLH122*, while *SrbHLH152* has collinear relationship with *SrbHLH91*, *SrbHLH126*, *SrbHLH141*, and *SrbHLH142*, respectively (Fig. 4, Table S2). These findings suggest that gene duplication events may be the primary cause of the expansion of the SrbHLH family.

### Correlation analysis between *SrbHLHs* and structural genes involved in RA biosynthesis

**Fig. 5:**
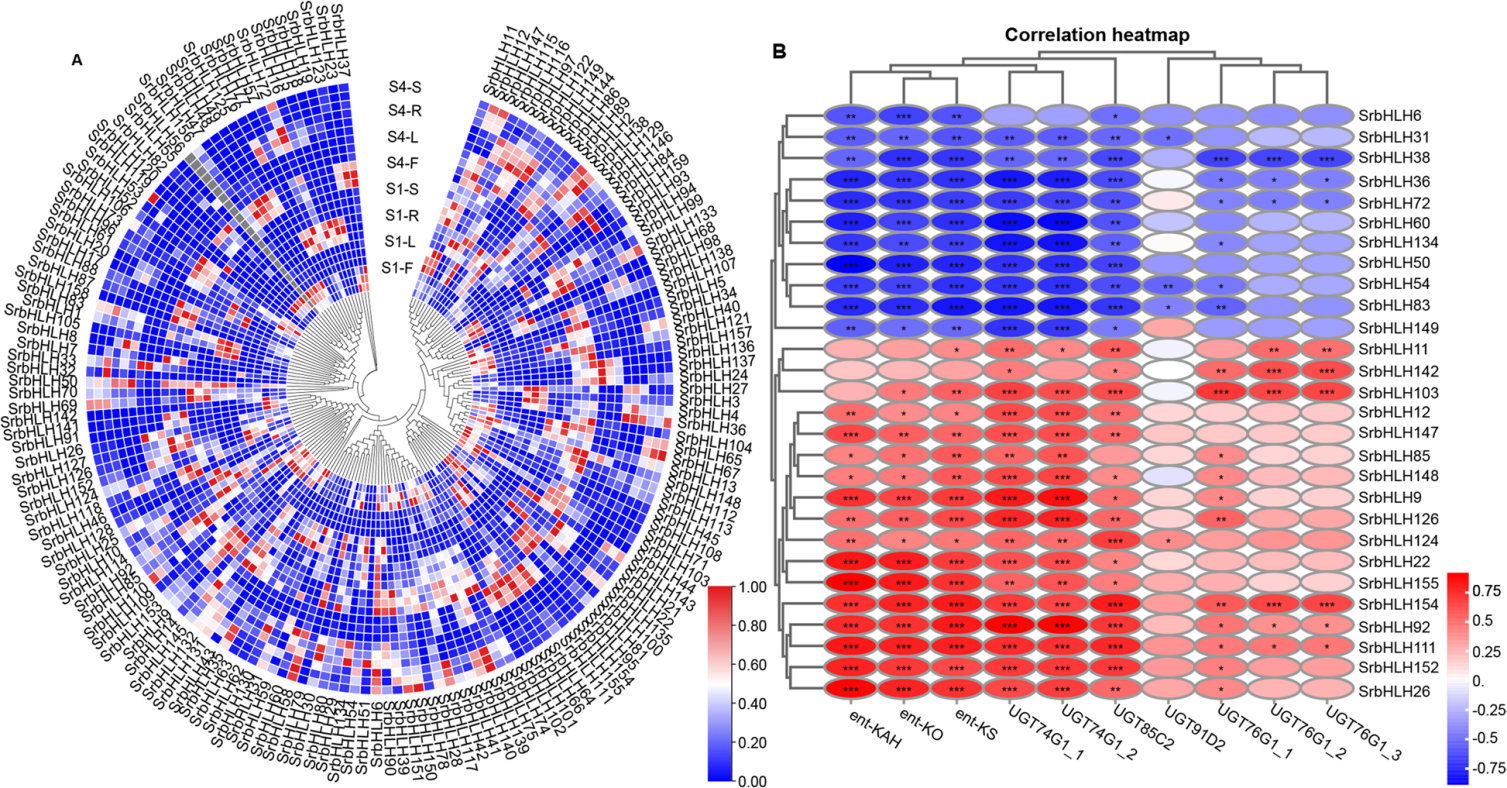
The expression patterns of *SrbHLH* genes and the correlation between the expression of *SrbHLHs* and structural genes involved in RA biosynthesis. **A** The expression patterns of *SrHLH* genes in different tissues of *S. rebaudiana*; **B** the co-expression relationship between *SrbHLHs* and structural genes involved in RA biosynthesis. Red circle: positively correlated; blue circle: negatively correlated. *, **, *** indicate a significant correlation at the 0.05, 0.01, or 0.001 level

Gene expression patterns provide valuable insights into gene function. As the content of SGs varies in different tissues of *S. rebaudiana* and the leaves are the main site of SG accumulation [16], we analyzed the expression of *SrbHLH* genes in different tissues. A heatmap was generated using Fragments Per Kilobase per Million (FPKM) values from RNA-seq data from two *S. rebaudiana* varieties (with higher RA content in S1 than S4) to show their tissue-specific expression patterns (Fig. 5A). Most *SrbHLH* genes exhibited tissue-specific expression patterns, such as *SrbHLH25*, *SrbHLH47*, *SrbHLH48*, and *SrbHLH49*, which were highly expressed in flowers, *SrbHLH111*, *SrbHLH154*, and *SrbHLH155*, which were highly expressed in leaves, *SrbHLH57*, *SrbHLH72*, and *SrbHLH76*, which were highly expressed in roots, and *SrbHLH69* and *SrbHLH70*, which were highly expressed in stems. Furthermore, some *SrbHLH* genes showed differential expression between the two varieties. For example, the expression level of *SrbHLH15*, *SrbHLH16*, *SrbHLH18*, and *SrbHLH19* in the roots of S1 was higher than that of S4, the expression level of *SrbHLH57*, *SrbHLH58*, and *SrbHLH88* in the flowers of S4 was higher than that of S1, and the expression level of *SrbHLH124* in the leaves of S1 was higher than that of S4 (Fig. 5A). These findings provide a basis for identifying bHLH transcription factors that regulate RA biosynthesis.

The biosynthesis pathway of RA in *S. rebaudiana* has been elucidated, with specificity mainly occurring in the later cyclization and modification pathways. Seven structural genes, including *ent-KS*, *ent-KO*, *ent-KAH*, *UGT74G1*, *UGT85C2*, *UGT91D2*, and *UGT76G1*, are involved in the formation of RA in *S. rebaudiana* [17, 23]. Using RNA-Seq data from different tissues of *S. rebaudiana*, co-expression analysis was conducted to identify essential *SrbHLH* genes regulating RA biosynthesis. Notably, we found that the expression levels of 11 *SrbHLH* genes were significantly negatively correlated with the seven structural genes, while the expression levels of 17 *SrbHLH* genes were significantly positively correlated with the seven structural genes (Fig. 5B). Therefore, we hypothesized that these 28 *SrbHLH* genes might be involved in regulating RA biosynthesis.

### *SrbHLH* gene expression patterns in various tissues.

**Fig.6:**
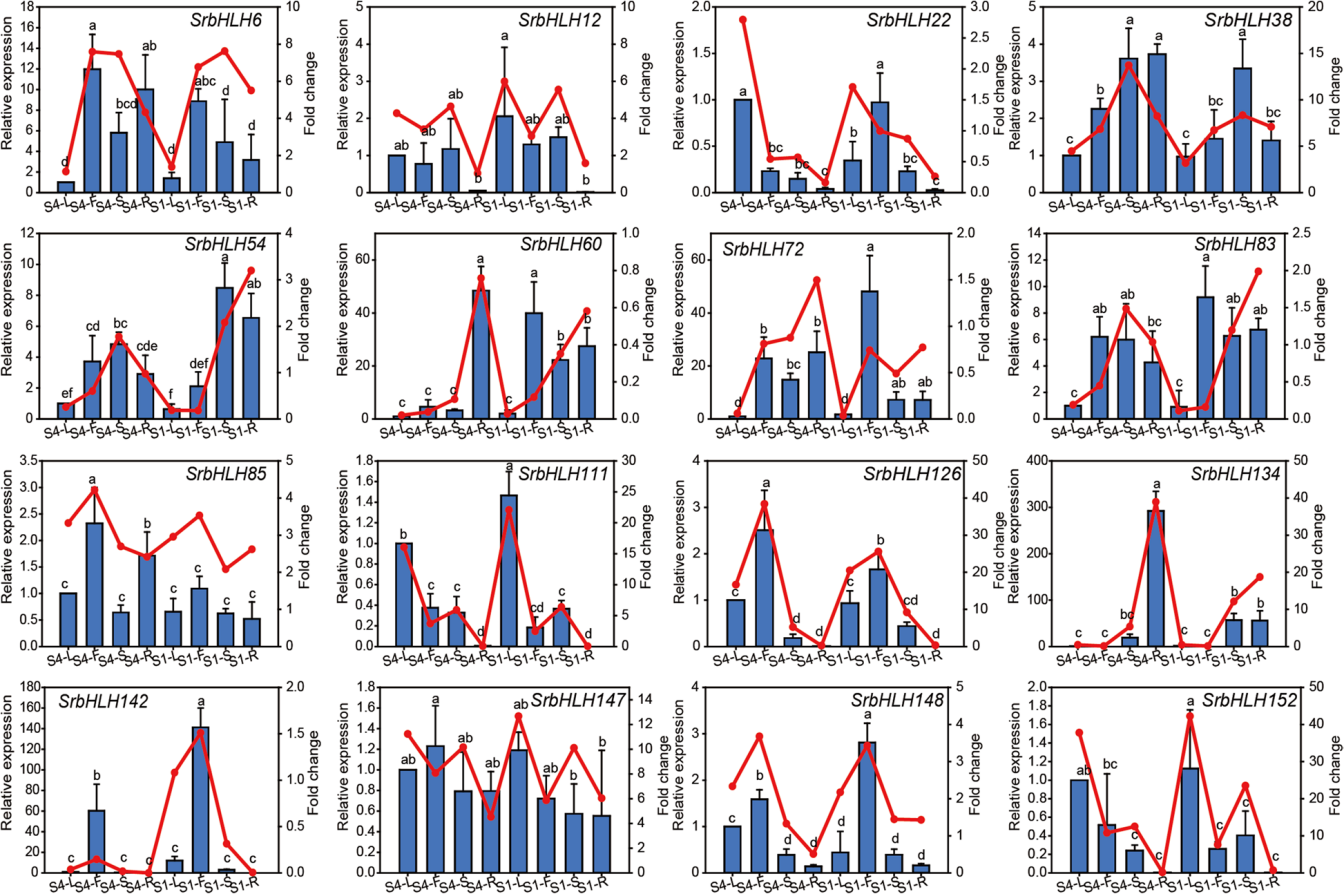
The expression patterns of candidate *SrbHLH* genes in different tissues of *S. rebaudiana* measured by qPCR

To validate the RNA-Seq results, we conducted qPCR assays to assess the gene expression of *SrbHLHs* in different tissues of *S. rebaudiana* varieties S1 and S4. We focused on 16 *SrbHLH* genes with the highest correlation with the critical RA biosynthesis genes *UGT74G1* and *UGT76G1.* As depicted in Fig. 6, the qPCR results were consistent with the RNA-Seq data. Notably, *SrbHLH22*, *SrbHLH111*, and *SrbHLH152* exhibited higher expression in leaves than other tissues, whereas *SrbHLH126*, *SrbHLH142*, and *SrbHLH148* showed higher expression levels in all tissues. Additionally, only *SrbHLH134* demonstrated significantly higher expression in the roots of variety S4 than that of other tissues. Overall, these results suggest that *SrbHLH22*, *SrbHLH111*, and *SrbHLH152* may have crucial roles in RA biosynthesis and accumulation.

### Expression of *SrbHLH* genes in response to MeJA treatment

**Fig. 7:**
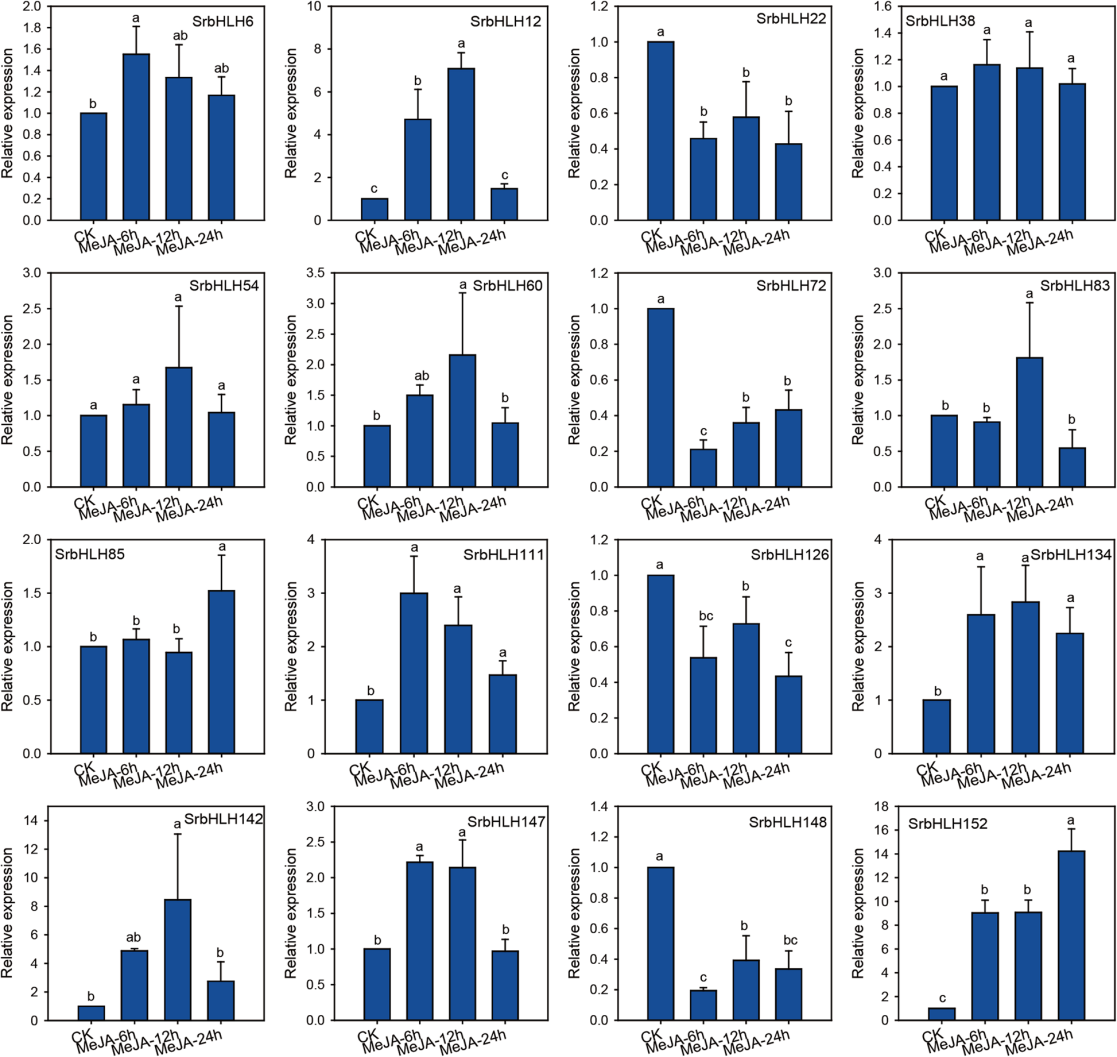
The expression patterns of candidate *SrbHLH* genes under MeJA treatment measured by qPCR

MeJA has been shown to regulate the biosynthesis of multiple secondary metabolites [24], such as flavonoid and soyasaponin biosynthesis in *G. uralensis* [25, 26]. Therefore, we examined the expression patterns of 16 *SrbHLH* genes selected in our study under MeJA treatment. As depicted in Fig. 7, all *SrbHLH* genes, except for *SrbHLH38* and *SrbHLH54*, were responsive to MeJA treatment. Among these 14 MeJA responsive genes, four SrbHLH genes (*SrbHLH22*, *SrbHLH72*, *SrbHLH128,* and *SrbHLH148*) were down-regulated, while ten SrbHLH genes (*SrbHLH6*, *SrbHLH12*, *SrbHLH60*, *SrbHLH83*, *SrbHLH85*, *SrbHLH111*, *SrbHLH134*, *SrbHLH142*, *SrbHLH147,* and *SrbHLH152*) were up-regulated under MeJA treatment. These results indicate that most of the *SrbHLH* genes are MeJA-responsive and may play a role in JA-regulated secondary metabolic processes.

### Functional verification of essential *SrbHLH* genes regulating rebaudioside A accumulation

**Fig. 8:**
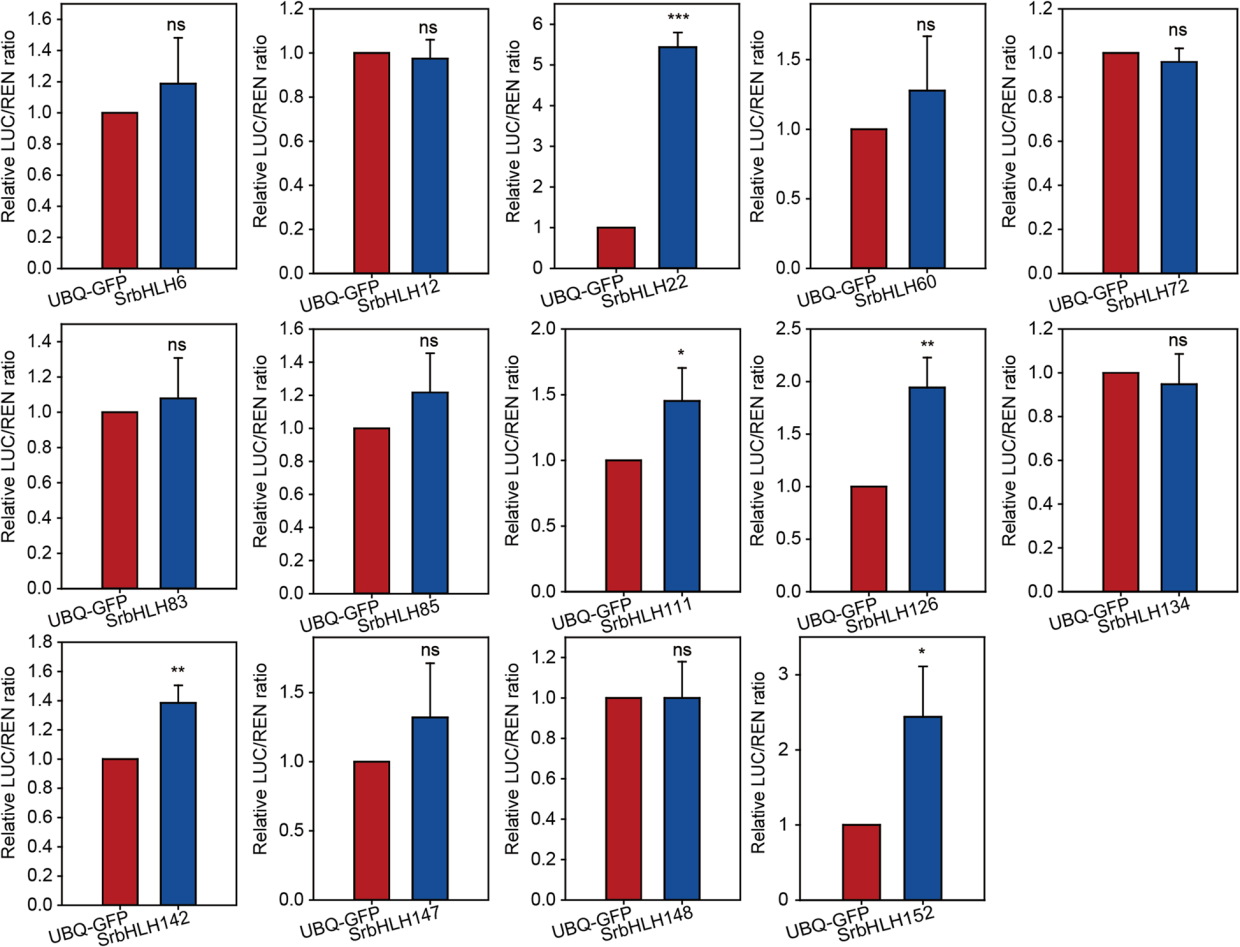
Transcriptional activation of *UGT76G1* by candidate SrbHLHs. The values shown are the means ± SD of the dual LUC/REN ratio. *, **, *** indicate a significant difference at the 0.05, 0.01, or 0.001 level

It is well established that *UGT74G1* and *UGT76G1* are two major genes involved in RA biosynthesis. UGT74G1 catalyzes the conversion of steviolmonoside to rubusoside, while stevioside is converted to RA under catalysis by UGT76G1 [17, 27]. In this study, we cloned and analyzed the transcriptional activation or repression of 14 selected MeJA reponsive *SrbHLH* genes on *UGT76G1* using a dual luciferase assay (DLA). Results showed that 5 SrbHLHs (SrbHLH22, SrbHLH111, SrbHLH126, SrbHLH142, SrbHLH152) transcriptionally activated the expression of *UGT76G1*, while SrbHLH6, 12, 60, 72, 83, 85, 134, 147, and 148 were implicated in regulating the expression of other structural genes involved in RA biosynthesis (Fig. 8).

### Subcellular localization of key bHLHs involved in regulating rebaudioside A accumulation

**Fig. 9:**
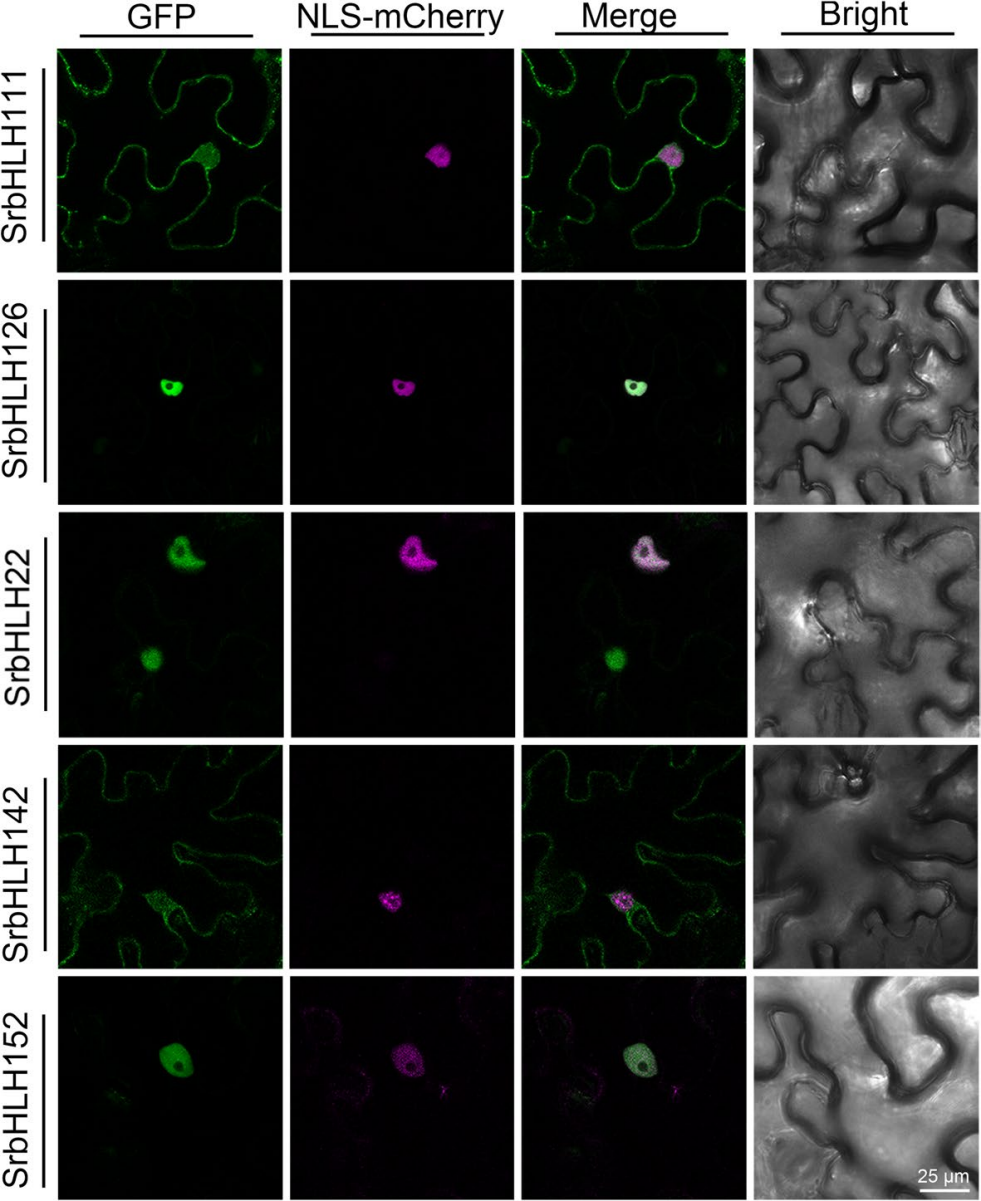
The subcellular localization of candidate SrbHLH proteins

Subcellular localization is a crucial factor in understanding gene function. Our prediction analysis revealed that SrbHLH proteins exhibit various subcellular localization patterns (Table 1). To further investigate this, we experimentally determined the subcellular localization of 5 SrbHLHs that regulate RA biosynthesis, as confirmed by DLA. Full-length cDNAs of each *SrbHLH* was fused to Green Fluorescent Protein (GFP) under the ubiquitin promoter and transiently expressed in *N. benthamiana* leaves. Our findings showed that SrbHLH111 and SrbHLH142 localized in both the nucleus and cytosol, while SrbHLH22, SrbHLH126, and SrbHLH152 were mainly localized in the nucleus (Fig. 9). These nuclear localization results were consistent with the bioinformatics prediction and the expected role of SrbHLHs as transcription factors.

## Discussion

The bHLH transcription factors (TFs) play crucial roles in plants, including response to abiotic/biotic stress and regulation of metabolic pathways. Nowadays, genome-wide characterization of *bHLH* genes have been reported in numerous plants. However, little is known about this gene family in *S. rebaudiana*, prompting us to systematically examine bHLH proteins in *S. rebaudiana*, especially looking into their function in regulating RA biosynthesis. Here, we identified 159 *SrbHLH* genes by investigating the *S. rebaudiana* genome, and based on classification methods from previous study [28], these 159 SrbHLH proteins were clustered into 18 groups. Generally, genes in the same branch had similar functions. Many SrbHLH members were proximal to the bHLH proteins in *Arabidopsis*, which were predicted to be homologous genes. For example, *AtbHLH* genes in subfamily N were responsive to the JA signal. These genes were reported to participate in the regulation of abiotic stress and terpenoid metabolism through JA-mediated pathways in plants [29, 30]. Thus, we predicted that SrbHLH proteins in subfamily N (including SrbHLH26, SrbHLH91, SrbHLH124, SrbHLH126, SrbHLH127, SrbHLH141, SrbHLH142, and SrbHLH152) also participate in the regulation of abiotic stress and terpenoid metabolism in *S. rebaudiana*. These phylogenetic findings will contribute to the prediction of functions of *SrbHLH* genes in *S. rebaudiana*.

The gene expression level is critically important for regulating plant biological progress. In general, genes with the same expression patterns are more likely to be involved in producing similar bioactive compounds [31]. Based on RNA-Seq data, this study revealed that 159 *SrbHLH* genes presented different expression patterns, and numerous tissue-specific *SrbHLH* genes were identified (Fig. 5A). These findings will contribute to the screening of candidate genes regulating RA synthesis. Interestingly, co-expression analysis using RNA-Seq data from different tissue samples demonstrated that 11 *SrbHLH* genes were significantly negatively correlated with the seven structural genes involved in RA biosynthesis, whereas 17 *SrbHLH* genes were significantly positively correlated with structural genes (*ent-KAH*, *ent-KO*, *ent-KS*, *UGT74G1*, *UGT76G1*, *UGT85C2*, and *UGT91D2*) (Fig. 5B), which were reported to the key critical genes for RA biosynthesis [17–19]. Among the 28 SrbHLH genes co-expressed with structural genes involved in RA biosynthesis, three SrbHLH genes (*SrbHLH22*, *SrbHLH111,* and *SrbHLH152*) detected by qPCR were highly expressed in the leaves of *S. rebaudiana*, which were the main tissues of RA accumulation. In addition, SrbHLH152 belongs to subfamily N, the same clade as AtbHLH genes that regulating response to abiotic stress and terpenoid metabolism pathways [29, 30]. Therefore, in the future, these co-expressed *SrbHLHs* with structural genes and highly expressed in leaves may be served as candidate transcription factors regulating RA synthesis.

Several studies have shown that MeJA treatment induces the accumulation of secondary metabolites in various plant species. For example, terpenoids were induced in *Catharanthus roseus* cells [32] and soyasaponins in *G. uralensis* [25]. MeJA treatment also affected the expression of critical genes in the biosynthesis of SGs in *S. rebaudiana* [28]. It is shown that MeJA increased the accumulation of SGs by acting on *UGT85C2* and *UGT76G1* gene expression [33]. This study identified 14 *SrbHLH* genes co-expressed with structural genes and responded to MeJA treatment. Among them, the expression of 4 *SrbHLHs* were down-regulated, and 10 were up-regulated (Fig. 7), suggesting their essential roles in JA-mediated biological processes, including RA biosynthesis. It is worth noting that bHLH proteins have been reported to regulate metabolic processes by interacting with other transcription factors or co-factors [34]. For example, the COI1/JAZs/MYC2 module has been identified as a core regulator of JA-mediated terpenoid accumulation in *Catharanthus roseus* [35]. Therefore, investigating the regulatory function of SrbHLHs and their interaction with co-factors in RA biosynthesis in future studies could be of great interest.

Transcription factors typically bind to specific *cis*-elements in the promoter region of target genes to regulate the synthesis of compounds catalyzed by the target genes by regulating their expression. In the case of the bHLH gene family, binding sites usually include G-box, E-box, or N-box. For example, CrMYCl or AaMYC2-like proteins have been shown to regulate the expression of structural genes by binding to the G-box of their promoter regions, affecting the biosynthesis of indole alkaloids or artemisinin [11, 12]. In the current study, we used DLA to examine the binding ability of candidate SrbHLHs to the promoter regions of structural genes involved in RA synthesis. The results showed that 5 SrbHLHs (SrbHLH22, SrbHLH111, SrbHLH126, SrbHLH142, SrbHLH152) transcriptionally activated the expression of *UGT76G1* (Fig. 8). Meanwhile, the expression pattern of these 5 selected *SrbHLHs* presented a positive correlation with that of *UGT76G1* and the other structural genes involved in RA biosynthesis (Fig. 5). Furthermore, the nuclear localization of these SrbHLH proteins were consistent with their expected role as transcription factors (Fig. 9). Therefore, these 5 SrbHLHs appear to be the most promising candidates for regulating RA synthesis. In the future, transgenic plants will be constructed to evaluate the function of these 5 SrbHLHs in the RA biosynthesis pathway.

## Conclusion

In this study, we identified 159 *bHLH* (*SrbHLH*) genes in the *S. rebaudiana* genome. We then constructed a phylogenetic tree to confirm the relationships between SrbHLH and AtbHLH proteins and found that SrbHLHs in the same group had similar protein motifs and gene structures. In addition, 263 pairs of segmental duplicated *SrbHLH* genes were identified, indicating that duplication events contributed to the expansion of SrbHLH family. Based on RNA-Seq data, we found that expression patterns of *SrbHLHs* differed across various tissues. Moreover, to identify candidate SrbHLHs which regulate RA biosynthesis, co-expression analysis between *SrbHLH* genes and structural genes involved in RA synthesis was conducted. The qPCR method analyzed the relative expression levels of the selected *SrbHLH* genes. Finally, SrbHLH22, SrbHLH111, SrbHLH126, SrbHLH142, and SrbHLH152 were confirmed as candidate regulators of RA biosynthesis through transient dual luciferase reporter assays (DLA) and subcellular localization analysis. These results may contribute to further understanding of the functions of SrbHLHs in SGs biosynthesis regulation and provide a theoretical basis for the application of *SrbHLH* genes in the molecular breeding of *S. rebaudiana*.

## Supplementary Information

**Additional file 1: Table S1.** Primers used in this study.

**Additional file 2: Table S2.** The collinear relationships among SrbHLHs.

## Data Availability

The datasets generated and/or analyzed during this study are available in NGDC (https://ngdc.cncb.ac.cn/) with accession no. PRJCA016649. Other data are available in the supplementary table.
